# Priority effects: How the order of arrival of an invasive grass, *Bromus tectorum*, alters productivity and plant community structure when grown with native grass species

**DOI:** 10.1002/ece3.6908

**Published:** 2020-10-16

**Authors:** Laura Weber Ploughe, Cameron N. Carlyle, Lauchlan H. Fraser

**Affiliations:** ^1^ Department of Natural Resource Sciences Thompson Rivers University Kamloops BC Canada; ^2^ Agricultural, Food, and Nutritional Science University of Alberta Edmonton AB Canada

**Keywords:** biomass, colonization history, competition, founder effects, relative growth rate, richness

## Abstract

Theories and models attempt to explain how and why particular plant species grow together at particular sites or why invasive exotic species dominate plant communities. As local climates change and human‐use degrades and disturbs ecosystems, a better understanding of how plant communities assemble is pertinent, particularly when restoring grassland ecosystems that are frequently disturbed. One such community assembly theory is priority effects, which suggests that arrival order of species into a community alters plant–plant interactions and community assembly. Theoretically, priority effects can have lasting effects on ecosystems and will likely be altered as the risk of invasion by exotic species increases. It is difficult to predict how and when priority effects occur, as experimental reconstruction of arrival order is often difficult in adequate detail. As a result, limited experimental studies have explored priority effects on plant community assembly and plant invasions. To determine if and how priority effects affect the success of invasive species, we conducted a greenhouse study exploring how the arrival order of an invasive grass, *Bromus tectorum*, affects productivity and community composition when grown with native grasses. We found evidence for priority effects, as productivity was positively related to dominance of *B. tectorum* and was greater the earlier *B. tectorum* arrived. This suggests that priority effects could be important for plant communities as the early arrival of an invasive species drastically impacted the productivity and biodiversity of our system at the early establishment stages of plant community development.

## Graphical abstract

We found evidence for priority effects, as productivity was positively related to dominance of *Bromus tectorum* and was greater the earlier *B. tectorum* arrived. This suggests that priority effects could be important for plant communities as the early arrival of an invasive species drastically impacted the productivity and biodiversity of our system at the early establishment stages of plant community development.

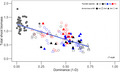

## 1 INTRODUCTION

The way in which plant communities assemble in a particular area has been debated for years and has generated several conceptual theories. While there are many community assembly theories, one theory that is regaining interest is the concept of priority or founder effects (henceforth “priority effects”). Priority effects explore how the arrival order of species into a community impacts plant–plant interactions and community assembly (Drake, 1991; Eriksson & Erikson, 1998; Fukami et al., 2016; Hess et al., 2019; Ke & Letten, 2018). Mechanisms driving priority affects appear to be a result of a combination of factors, including, the ability of early‐arriving species to reduce resources (nutrients, water, space, and light) preventing colonization from later arriving species (Fargione et al., 2004; Fukami, 2015; Vannette & Fukami, 2014; Werner et al., 2016). These priority effects can have lasting effects on diversity, plant composition, and soils (biological, chemical, and physical properties), often resulting in altered ecosystem function (Chase, 2003; Fukami, 2015; Hess et al., 2019; Ke & Letten, 2018). However, it is difficult to predict how and when priority effects occur, as arrival order is often difficult to reconstruct in adequate detail (Fukami, 2015; Fukami et al., 2016; Hess et al., 2019).

Priority effects and other community assembly theories provide conceptual foundations for understanding why particular species form a community in a particular location (Chase, 2003; Keddy, 1992) and have been historically explored through models, such as Lotka‐Volterra competition models (Ke & Letten, 2018; Lewontin, 1969; May, 1971). Field and greenhouse studies examining priority effects have been implemented to a much lesser extent (Fukami et al., 2016; Hess et al., 2019), although more studies are being conducted (i.e. EJRNÆS et al., 2006; Grman & Suding, 2010; Sarneel et al., 2016; Vaughn & Young, 2015). These empirical studies have reported that providing even a short time advance for native species to grow, as little as one or a few weeks, can decrease plant invasion and increase native species success (Firn et al., 2010; Grman & Suding, 2010; Vaughn & Young, 2015).

Understanding how and why communities assemble and the role that plant–plant interactions have on assembly is becoming increasingly important as communities experience changes to local climate, such as increases in the frequency and intensity of drought (He et al., 2013; Ploughe et al., 2019), land‐use change (Houghton & Nassikas, 2017), and other disturbances (Chase, 2003), which can all result in increases in biological invasions and severely alter plant community assembly and function (Hess et al., 2019; McNeely, 2006). This is particularly relevant for heavily disturbed and invaded land, which are often more difficult to successfully restore. Grassland ecosystems are of particular concern, which are being degraded rapidly through a variety of anthropogenic activities, are often heavily invaded as a result of these disturbances, and are one of the most sensitive ecosystems to altered climate (Newman et al., 2014; Parks et al., 2005; Prevéy & Seastedt, 2014; Rinella et al., 2016). In semiarid grasslands, degradation can lead to sites that are dominated by annual forbs and grasses, including *Bromus tectorum*, an early‐seral annual species (Briske et al., 2005; Newman et al., 2014; Suding et al., 2004). The use of ecological theory, such as priority effects, may be one technique to introduce more natives and fewer invasive species, improving restoration methods (Hess et al., 2019; Ke & Letten, 2018).

As a result of the limited experimental studies exploring priority effects on community assembly and plant invasions and to obtain greater control over the experiment, we conducted a greenhouse study that explores how the arrival order of an invasive grass, *Bromus tectorum* (cheatgrass), affects productivity and community composition when grown with native, perennial grasses from western North America. *Bromus tectorum* is found throughout Canada and the United States and is considered of high concern, particularly in northwestern North America, as it can completely replace native vegetation (Clinton et al., 2010; Mack, 1981; Upadhyaya et al., 1986). Additionally, *B. tectorum* is an agricultural and rangeland pest (Morrow & Stahlman, 1983) and increases wildfire risks, as dry *B. tectorum* is highly flammable (Clinton et al., 2010; Mack, 1981; Upadhyaya et al., 1986). *Bromus tectorum's* phenology is timed to precipitation events (Mack, 1984; Perkins & Hatfield, 2014), and while the species generally germinates in the fall or winter, its germination may be staggered from August to May and may occur in a series of pulses within a few days after a precipitation event (Carpenter & Murray, 2005; Kaczmarski, 2000; Mack, 1984). This ability to germinate early and at various times during the growing season as well as *B. tectorum's* ability to reach maturity earlier than native species could allow its dominance within communities (Clinton et al., 2010; Kaczmarski, 2000; Monsen, 1992; Zouhar, 2003). *Bromus tectorum* can have a high competitive advantage over native species, which is likely a result of its growth early in the spring and rapid growth response to seasonal water availability (Perkins & Hatfield, 2014), but it is unclear how these priority effects will affect community dynamics.

Here, we explored how priority effects alter plant community composition by examining the effects that the timing of arrival of *B. tectorum* had on productivity and dominance when planted with three North American native, perennial grasses. Our objectives were to assess the impact of the arrival order of *B. tectorum* on aboveground productivity when grown with native grasses, to explore how priority effects impacted community structure, specifically, evenness, and dominance, when native grasses are grown with an exotic grass, *B. tectorum*, and to explore a possible relationship between productivity and dominance. We hypothesized that *B. tectorum* would impose priority effects, as a result of *B. tectorum's* ability to germinate and grow rapidly following a precipitation event (i.e., watering), specifically reduced growth in the other species and increased dominance of *B. tectorum* when grown earlier than native grasses. Since the native grasses tend to have slower growth rates, we expected the likelihood that native grasses would impose priority effects would increase the later that B. tectorum arrives by limiting resources, such as light, space, water, and nutrients.

## 2 METHODS AND MATERIALS

### 2.1 Study species

Four species were used in the experiment: *Bromus tectorum*, *Festuca campestris*, *Poa secunda*, and *Pseudoroegeneria spicata*. All seeds were collected in Lac du Bois Protected Grasslands Area in British Columbia, Canada under Park Use Permit #102724. The three native species were selected, because these are common species within the bunch grass biogeoclimatic zone within the Lac du Bois grassland, where *B. tectorum* is invasive (BC Parks 2015; Fritch, Sargent, Mackenzie, Delesalle, & Delesalle 2009). These species are important for maintaining native diversity in these grasslands and are important forage for wildlife and cattle (Church et al., 2015). *Bromus tectorum* is an exotic, annual species that was introduced into North America from Europe in the 18th century and has spread throughout most of the continent (Upadhyaya et al., 1986; Valliant et al., 2007). Multiple genotypes of *B. tectorum* indicate unique introduction events of this species through multiple entry ports in eastern and western Canada (Valliant et al., 2007). Although genetic variation of the species is relatively low, phenotypic plasticity is high in *B. tectorum*, making the species adaptable to many environmental conditions (Valliant et al., 2007). *Bromus tectorum* is considered a noxious weed, particularly in rangelands, where it tends to germinate earlier than native species and can extract moisture from shallow soil layers inhibiting the establishment of desirable perennial native species (Upadhyaya et al., 1986), such as those in this study: *F. campestris*, *P. secunda*, and *P. spicata*.

### 2.2 Experimental design

The experiment was completed at the Research Greenhouse at Thompson Rivers University in Kamloops, British Columbia, Canada. Greenhouse conditions were controlled electronically (Argus control system) to maintain daytime conditions at 22°C and 60% relative humidity and nighttime conditions at 15°C and 85% relative humidity. A 14:10 hr day:night cycle was established using supplemental lighting supplied by three 1,000 W halogen overhead lamps. Seeds of each species were geminated in Petri dishes on a damp layer of sand and were transplanted into pots at four planting intervals (every 2 weeks). Each species had a radicle length of at least 15 mm and a single leaf before seedlings were transferred into 4‐inch plastic pots lined with landscape fabric to prevent sand from leaking; each pot was filled with approximately 1 L of clean builder's sand (King Play Sand©). Pots were regularly bottom‐watered with rain water to maintain 5 mm of water at the base and received 100 ml of Rorison's solution every seven days (Hendry et al., 1993).

Seedlings of each species were introduced to a pot at an interval of one species per planting interval without repeating a species until four individuals were in the pot, resulting in 24 possible combinations of arrival orders. Four individuals of the same species were also planted at the same planting intervals to serve as a control, that is, intraspecific competition control. An additional control was also included where an individual plant of each species was planted alone at each of the four planting intervals, that is, no competition control. All treatments were replicated six times into blocks. Seedlings were checked 3 days after transplant, and mortalities were replaced with individuals, which had been planted individually in a separate pot at the time of each planting. Seedlings that died after 3 days were counted as mortalities.

By the end of 6 weeks, the time of the fourth planting interval each pot contained four individuals of each species and only the order of arrival varied among the pots; intraspecific competition control pots had four individuals of the same species, and no competition control pots had only one individual. After 10 weeks, shoots from all individuals were separated from the roots and cleaned of sand. Shoots, hereby, aboveground biomass, were oven dried at 65°C for 48 hr and weighed to 5 decimal places to assess the effects of arrival order on productivity, evenness, and dominance. Richness (S) was considered the number of species in the pots after 10 weeks. To better understand the relative abundance of each species, evenness was calculated using Pielou's evenness (J), where J = H′/log(S) (Oksanen et al., 2016). H′ is the Shannon–Weaver diversity index and was calculated using the equation, Σp_i_ln(p_i_), where p_i_ is the proportion of species. We also calculated the relative intensity index (RII) for each species at each time interval to better understand how plant interactions influenced productivity (Armas et al., 2004). RII represents the competitive effect of intra‐ and interspecific interactions compared with when the species is grown alone and is calculated using the formula: RII = (B_w_ – B_0_)/(B_w_ + B_0_), where B_w_ is the biomass of a species growing with either type of competition and B_0_ is the biomass of a species that is grown without competition. A negative effect indicates the species had a competitive effect and a positive effect indicates the species had a facilitative effect on the other individuals or species (Armas et al., 2004).

### 2.3 Data analysis

Data were analyzed using mixed model of analysis of variance in R version 3.5.2 ‐ "Eggshell Igloo" (R Core Team, 2013) using the “lmer” function from the package “lme4” (Bates et al., 2015; R Core Team, 2013). All models included block (replicate) as a random variable. Models regarding total shoot biomass included both intra‐ and interspecific competition treatments. To assess how competition from the various species affected productivity, a model was created using RII as the response variable and the four planting intervals, four species, and two competition levels (intraspecific, and interspecific) were used as explanatory variables. Models exploring plant composition were also created using all relevant response variables (percent biomass by species, richness, dominance, and evenness) but included only interspecific competition treatments. Models were selected based on meeting model assumptions, and quality was tested using the Akaike information criterion (AIC); percent biomass by species was natural log transformed for normality. Tukey's HSD post hoc analyses were performed on all models using the “emmeans” package to generate estimated marginal means (EMMs; also known as least‐squares means; Lenth, 2019).

## 3 RESULTS

### 3.1 Productivity

**Figure 1:**
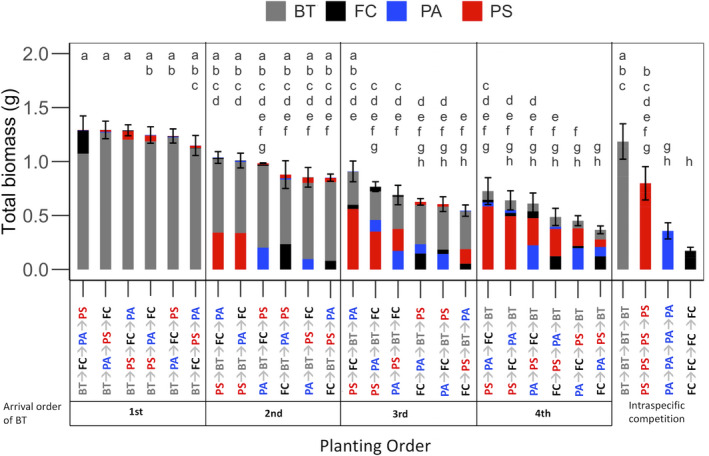
Aboveground productivity (total shoot biomass) of the 28 planting order treatments according to interspecific competition in order of productivity and the arrival time of *Bromus tectorum* and intraspecific competition. Each bar represents the mean of the total shoot biomass with standard error. Letters represent statistically similar groups (Tukey's HSD, alpha = 0.05). The four species planted, BT, *Festuca campestris* (FC), *Pseudoroegeneria spicata* (PS), and *Poa secunda* (PA), are color coded as gray, black, red, and blue, respectively. The proportion of each species contribution to aboveground productivity is stacked within each bar according to arrival order

We found that the planting order altered aboveground productivity (*F*_27,129.2_ = 13.7; *p* < .0001). As predicted, the treatments where BT was the first species to arrive had the greatest productivity (*p* < .05, Tukey's HSD; Figure 1). Productivity was reduced slightly or was similar to treatments when BT was the first or second species to arrive and generally decreased when BT was the third or fourth species planted (*p* < .05, Tukey's HSD). The treatments with the lowest aboveground productivity were *Festuca campestris* (FC) grown with conspecifics (FC → FC → FC → FC), which was similar to *Poa secunda* (PA → PA → PA → PA) grown with conspecifics and the treatment with the planting order of *Poa secunda* (PA), FC, *Pseudoroegeneria spicata* (PS), then BT (PA → FC → PS → BT; *p* < .05, Tukey's HSD).

**Figure 2:**
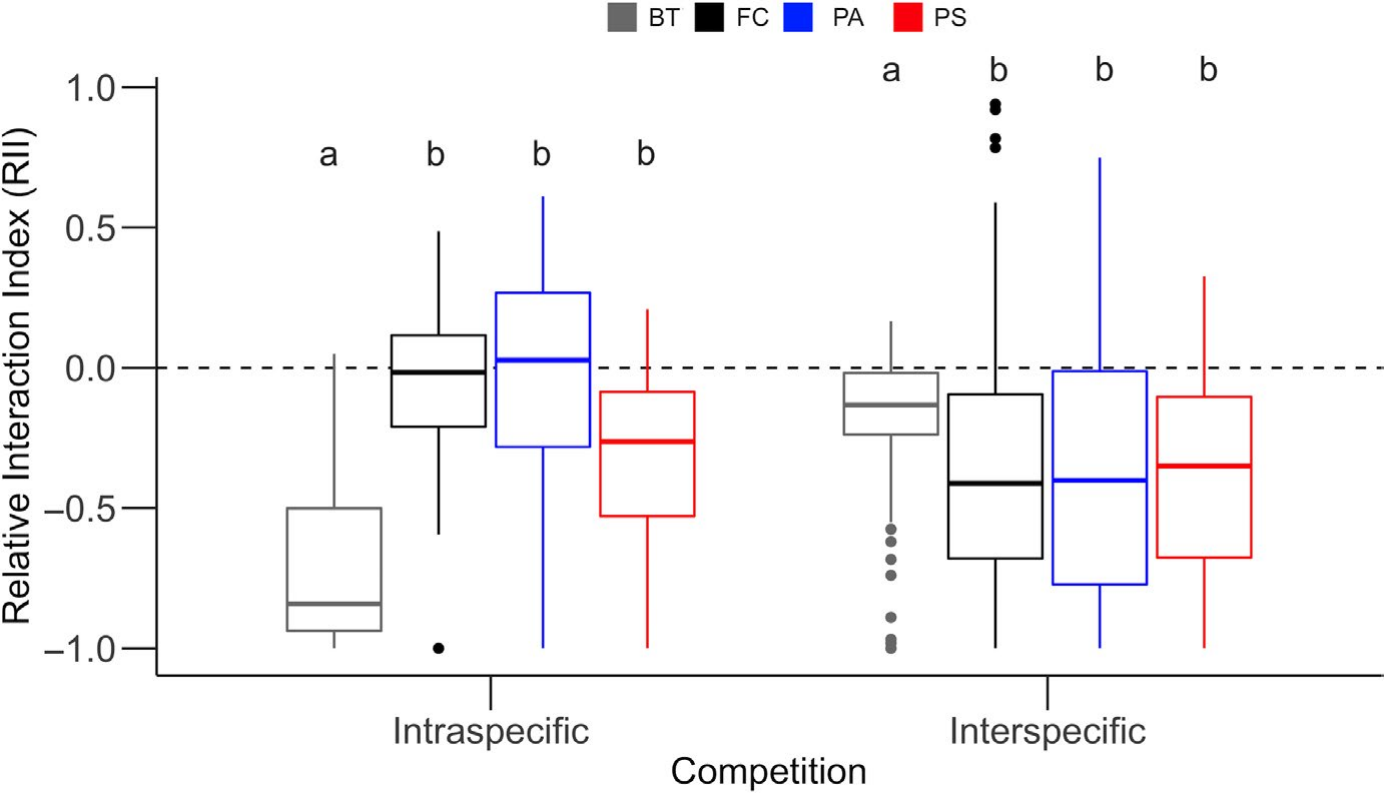
Relative interaction index (RII) of species in relation to the level of competition (intra‐ and interspecific). The four species planted, *Bromus tectorum* (BT), *Festuca campestris* (FC), *Pseudoroegeneria spicata* (PS), and *Poa secunda* (PA), are color coded as gray, black, red, and blue, respectively. Boxes represent the interquartile ranges (25th percentile and 75th percentile) and the line within each box indicates the median. The whiskers represent the spread of the data that falls. Points represent outliers from this spread or values 1.5 times greater or less than the inner quartile range. Letters indicate the significant difference between species within the same type of competition (intra‐ or interspecific competition)

The competitive interactions influencing the productivity of the species (RII) were impacted by the type of competition (intra‐ or interspecific) and by species (*F*_3, 614_ = 22.8, *p* < .0001), but the arrival time of the species did not have an impact on competition. Under interspecific competition, all three native grasses were more competitive than *B. tectorum* (BT), but under intraspecific competition, *B. tectorum* (BT) was more competitive than all three native grasses (Figure 2; Tukey's HSD, *p* < .05). *Bromus tectorum* (BT) was more competitive when grown with conspecifics compared to growing with native grasses (Tukey HSD, *p* < .05). *Festuca campestris* (FC) and *P. secunda* (PA) were more competitive when grown under interspecific competition, and *P. spicata* (PS) demonstrated similar competitive intensity in both intra‐ and interspecific conditions (Tukey HSD, *p* < .05).

### 3.2 Plant composition

**Figure 3:**
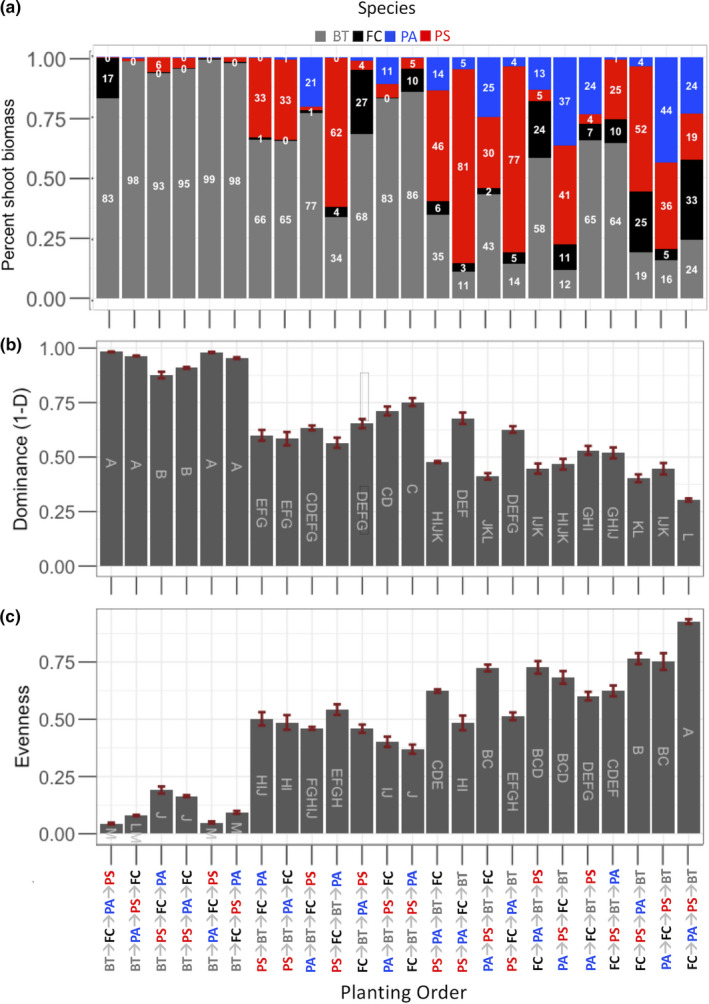
Plant composition of the 24 interspecific treatments arrange in order of highest to lowest productivity (a) Mean percent of species shoot biomass, shown as numbers within each bar. Missing values are less than 2 or zero. Bars are stacked in the order of species (*Bromus tectorum* (BT), *Festuca campestris* (FC), *Pseudoroegeneria spicata* (PS), and *Poa secunda* (PA)) and are color coded as gray, black, red, and blue, respectively. (b) Mean dominance (1‐D) and (c) evenness (J) with standard error. Letters represent statistically similar groups (Tukey's HSD, alpha = 0.05)

We explored how order of arrival affected plant composition in terms of the proportion that each species contributed to aboveground biomass in each interspecific competition treatment, exploring richness, dominance, and evenness. We expected that the proportion of BT would be highest the earlier it was planted and that this would lead to reductions in richness and evenness and increases in dominance. The proportion that a species contributed to total biomass was affected by the interaction between the planting order and species (*F*_69, 460_ = 21.66; *p* < .0001; Figure 3a). BT contributed the largest proportion to shoot biomass when it was the first species or second species planted, regardless of the planting order of the other species (*p* < .05, Tukey's HSD). PS contributed the largest proportion or similar to biomass as BT when BT was planted third or fourth in the following treatments: PS → FC → BT → PA; PS → PA → BT → FC; PS → PA → FC → BT; BT → PS → PA → FC & PS → FC → PA → BT. BT contributed a lower proportion to shoot biomass in some treatments when FC or PA was the founder species and BT was the last species planted: PA → PS → FC → BT; FC → PS → PA → BT; PA → FC → PS → BT; & FC → PA → PS → BT (*p* < .05, Tukey's HSD; Figure 3a).

**Figure 4:**
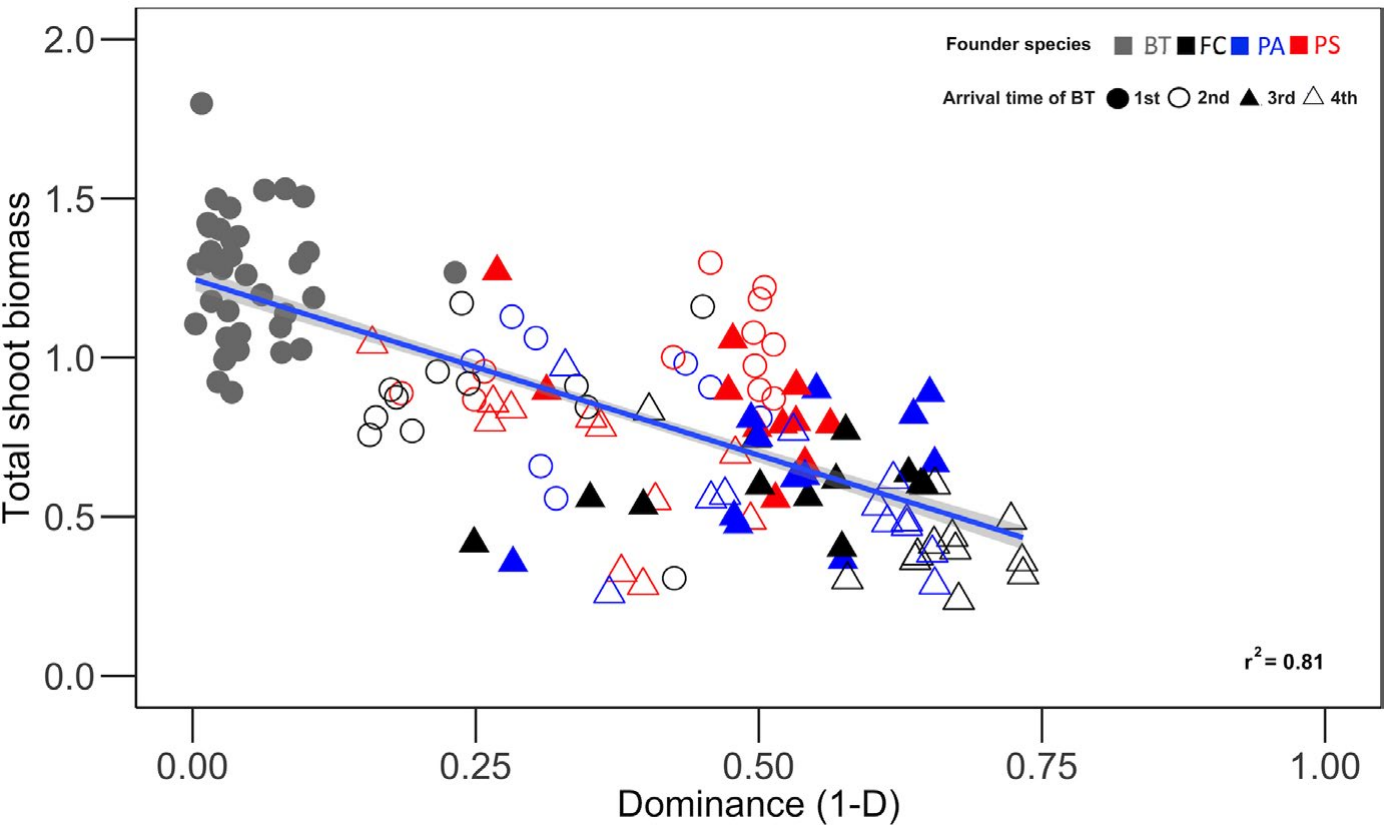
Linear relationships between the shoot biomass (g) and dominance (1‐D), where 0 indicates high dominance and low diversity and 1 indicated high diversity. Individual points represent each pot, which are color coded according to the first species planted or founder species (BT = *Bromus tectorum*, FC = *Festuca campestris*, PS = *Pseudoroegeneria spicata*, and PA = *Poa secunda*). The colors represent the first species planted, where BT = gray, FC = black, PA = blue, and PS = red. The shapes correspond to the order in which BT was planted: 1st = filled circle, 2nd = open circle, 3rd = filled triangle, and 4th = open triangle

Although planting order affected richness statistically (*F* = 1.67_23, 110_; *p* = .04), further analysis did not reveal differences between treatments (Tukey's HSD, alpha = 0.1). Planting order altered both dominance and evenness (*F*_23, 110_ = 52.69 & *F*_23,115_ = 32.77, respectively; *p* < .0001). We found that dominance was generally higher when BT was planted first or second and was reduced when BT was planted later (Tukey HSD, *p* < .05; Figure 3b). Conversely, evenness was lowest when BT was the founder species and increased when BT arrived later (Tukey HSD, *p* < .05; Figure 3c). Both evenness and dominance were found to be linearly related to shoot biomass and planting order (*F*_23,506_ = 9.60 & *F*_23,506_ = 9.23, respectively, *p* < .0001; Figure 4).

## 4 DISCUSSION

This experiment demonstrates that priority effects can impact both productivity and composition of grasses within a mesocosm setting, and the species that demonstrated the largest impacts on productivity and community structure was *Bromus tectorum*, an aggressive, invasive species in North American grasslands. Simply by altering arrival of only four plant species that commonly co‐occur in western North American, semiarid grasslands, we found that the aboveground productivity of plant communities grown with *B. tectorum* can vary by a factor of 3.25 over a 10‐week growth period. Considering the important relationships between productivity and other ecosystem properties such as biodiversity (Loreau et al., 2001), it is clear that assembly sequence should be an important consideration in our understanding of plant communities and ecosystem processes, especially in annual communities and early successional stages. Further, this will be particularly important to consider when restoring sites that have been invaded with *B. tectorum*, which may experience *B. tectorum* germination in a series of pulses following rainfall events from August through May (Carpenter & Murray, 2005; Kaczmarski, 2000; Mack, 1984).

Specifically, we found that earlier arrival of *B. tectorum* increased total aboveground productivity when grown with native grass species. We suspect that this effect was the result of *B. tectorums*' rapid growth compared with the native perennial grasses, and this species' ability to alter its phenotypic expression depending on environmental conditions (Valliant et al., 2007). In this case, conditions were highly suitable for plant growth, and it would be interesting to explore whether or not the same effects would be found when *B. tectorum* is grown under drier and nutrient poor conditions. Our experiment further revealed that the competitive effect (RII) of *B. tectorum* was reduced when grown with native grasses, suggesting that the native plants could have an advantage if *B. tectorum* arrives to the community later and is not already a monoculture at the site being restored.

Dominance was impacted by the order of species arrival in this study, and *B. tectorum* dominance was positively correlated with productivity and early arrival within the community. Treatments where *B. tectorum* was the founder species had the highest productivity and dominance, as the other species contributed little to no aboveground biomass. Dominance was reduced significantly when *B. tectorum* arrived last, as plant composition became more even. In the case of severely disturbed sites, where succession and priority effects will impact plant assembly and succession, the presence of *B. tectorum* early in community assembly will likely result in its dominance. These differences in plant composition can have large effects on ecosystem processes if traits of one or a few species dominate (Hooper & Vitousek, 1997). Monocultures of *B. tectorum* can disrupt ecosystem function through losses to biodiversity and plant traits specific to this species (Dukes & Mooney, 2004; Hooper & Vitousek, 1997). Specifically, *B. tectorum* has been found to alter nutrient cycling (Blank, 2008; Evans et al., 2001; Knapp, 1996; Weidenhamer & Callaway, 2010), hydrologic regimes, and fire frequency (Balch et al., 2013; Bradley et al., 2018; Brooks et al., 2006; Dyer & Rice, 1999; Levine et al., 2003).

Our experiment investigated colonization and early establishment phases of plant community development, as the study was terminated after 10 weeks. And, although *B. tectorum* dominated biomass as the founder species as well as the second order arrival it is not known whether *B. tectorum* dominance would persist for more than one generation; *B. tectorum* was the only annual species in this study but is a prolific seed generator. However, given that *B. tectorum* has been shown to alter soil properties, such as nitrogen dynamics (e.g., Evans et al., 2001) and increase prevalence of wildfire (e.g., Knapp, 1996), our study provides important information on conditions that favor dominance, namely when *B. tectorum* was planted early in succession and was able to take advantage of earlier and faster growth. Seedling emergence and germination are crucial events in the life cycle of plants, as the time at which germination or emergence occurs often determines subsequent performance and success particularly under competitive situations (Harper, 1977; Verdu & Traveset, 2003). Here, we also found that even short delays in seedling emergence time can impact the fitness of perennial native grass species when grown in the presence of an aggressive annual species.

This experiment explored how the impacts of both priority effects and invasion of *B. tectorum* in relation to native species establishment and success during early successional phases of plant communities. Thus, if a site is at risk or already invaded by *B. tectorum*, restoration methods should take this into account. Our study appears to support the idea that the phenology of *B. tectorum* may be a contributing factor to its invasion throughout North America, as *B. tectorum* tends to grow early in the season compared with native species. Shifting precipitation patterns resulting from global climate change will influence the success of *B. tectorum* and other non‐native winter annuals (Prevéy & Seastedt, 2015). As temperatures increase, precipitation in late winter and early spring will continue to shift from snow to rain (Zhang et al., 2019) and increases in rain early in the growing season could benefit early‐growing winter annuals, such as *B. tectorum*, to the detriment of native species (Prevéy & Seastedt, 2015). Our study confirms that early arrival of *B. tectorum* can have major implications for community dynamics and productivity. The risk of invasion by this species may increase under future climate scenarios, suggesting that future studies are need to understand the arrival order of species will be influences by altered abiotic conditions.

Therefore, it will be particularly important in restoration studies and land management to either facilitate the establishment of native species first, restrict early establishment of *B. tectorum*, or both. Further, this demonstrates a need for further exploration of the role of priority effects in species invasion and community assembly, particularly under increased globalization and changes in climate, resulting from climate change (Hess et al., 2019; Ke & Letten, 2018; Ploughe et al., 2019). As previously mentioned, grasslands are being degraded rapidly, and to effectively reverse or slow this reduction, we must have a better understanding of how community's assembly. The order in which species arrive, priority effects, may be one reason for alternative stable states, such as those dominated by *B. tectorum*, that are resistant to restoration.

## CONFLICT OF INTEREST

The authors do not have no known competing financial interests or personal relationships that could have appeared to influence the work reported in this manuscript.

## AUTHOR CONTRIBUTIONS

**Laura Ploughe:** Formal analysis (equal); investigation (equal); visualization (lead); writing–original draft (lead); writing–review and editing (lead). **Cameron Carlyle:** Data curation (lead); methodology (lead); writing–review and editing (supporting). **Lauchlan Fraser:** Supervision (lead); writing–review and editing (supporting).

## Data Availability

Data will be available on FigShare https://doi.org/10.6084/m9.figshare.12469091.
